# Traumatic brain injury from a developing country in the CT-scan era: a 13-year prospective in-hospital cohort study

**DOI:** 10.3389/fneur.2026.1889484

**Published:** 2026-10-26

**Authors:** A. O. Adeleye, O. O. Agboola, T. A. Oyemolade, T. A. Malomo, A. L. Azeez, T. B. Rabiu, A. K. Ukachukwu

**Affiliations:** 1 Division of Neurological Surgery, Department of Surgery, College of Medicine, University of Ibadan, Ibadan, Nigeria; 2 Department of Neurological Surgery, University College Hospital, Ibadan, Nigeria; 3 Division of Neurosurgery, Department of Surgery, Federal Medical Center Owo, Owo, Nigeria; 4 Department of Neurosurgery, Spencer Fox Eccles School of Medicine, University of Utah, Salt Lake City, UT, United States; 5 Department of Surgery, Osun State University, Osogbo, Nigeria; 6 Department of Neurosurgery, Duke University Health System, Durham, NC, United States; 7 Duke Global Neurosurgery and Neurology, Durham, NC, United States

**Keywords:** Africa, computed tomography, developing country, low middle-income country, Nigeria, traumatic brain injury

## Abstract

**Introduction:**

Most previous reports from Africa and other low middle-income countries (LMIC) on cranial neurotrauma address a more loosely defined subject of head injury (HI), rather than traumatic brain injury (TBI), and lack deployment of standardized scoring systems. We present a prospective TBI cohort from an academic neurosurgery practice at Ibadan, Nigeria, the nation's foremost university teaching hospital where TBI constitutes a core component of our clinical activity.

**Materials and methods:**

This was a prospective cohort study of patients enrolled in a TBI registry over 13 years. We analyzed the clinical epidemiology of TBI, defined as cranial trauma with definite perturbation of brain function, qualified/quantified using relevant clinical features, the Glasgow Coma Scale (GCS), the Abbreviated Injury Scale-Head (AIS-Head), and Injury Severity Score (ISS). Brain imaging was graded using the Rotterdam CT scoring system. In-hospital outcome was assessed with Glasgow Outcome Scale (GOS), dichotomized for analysis.

**Results:**

There were 1,440 TBI cases, 1,110 (77.1%) males, mean age [32.2 Standard deviation (SD), 19] years. Most (76%) were referrals, with delayed presentation from outlying health facilities, median duration 13 [interquartile range (IQR) 5, 48] hours. Road trauma accounted for 1,173 (82%), even in the elderly, predominantly by two- and three-wheelers (57%); fall for 136 (9.4%), this mainly in childhood. Clinically, loss of consciousness (LOC) occurred in 89%, median duration 11.0 (IQR 2, 48) hours, vomiting 25%, seizures 16%, and anisocoria in 27%. In 86% the AIS-head was >2; the TBI was moderate-severe, GCS 3–12, in 47.0%. Brain CT was obtained in 1,046/1,440 (72.6%), while 13% received acute operative treatment. In-hospital mortality was 21%, mostly among the young and the severely injured. Good outcome (GOS: good recovery/moderate deficit) was 75%. The outcomes were significantly associated with the TBI severity per the GCS scores and brain CT severity.

**Conclusions:**

The clinical epidemiology of TBI from this single-institution LMIC series differs substantially from that of high-income countries. A 21% mortality predominantly affecting young economically productive adults represents a serious public health burden. Substantial gaps persist across many aspects of the TBI-management continuum, presenting clear opportunities to improve the current sub-optimal outcome-metrics in sub-Saharan Africa.

## Introduction

Traumatic brain injury (TBI) exacts its greatest toll in the world's low middle-income countries (LMICs) (1, 2). Unlike in many Western countries where fall now constitutes the leading mechanism of TBI (3, 4), road trauma remains the main culprit in most LMICs, particularly in Africa (5–8). The African region (per the World Health Organization global regional designation) carries a particularly paradoxical burden of road traffic deaths: it accounts for up to 19% of global road traffic fatalities despite comprising only 15% of the World's population, and a mere 4% of its registered road vehicles (5, 9). These road-trauma fatalities are predominantly attributable to TBI in major proportions of most published series (10).

This is an existential paradox that is further heightened by the fact that the same African LMICs with very high proportions of the global TBI-burden, 35% of all trauma admissions in one study from Benin Republic (11), actually have only relatively negligible contribution to the global literature on the subject. In one review, publications on TBI from Africa were found to constitute only 1.1% of the global materials, compared with 51.0% from the United States of America and Canada, and 25% from Europe (12). Another review within this decade also showed that even within sub-Sahara Africa (SSA), the overwhelming proportions of TBI publications (75%) came only from South Africa (5).

Even so, the few and far between studies touching on the subject from SSA also present some methodological issues. They predominantly report only epidemiological and socio-demographic data. They fall short of rigorous data analysis by not deploying standardized scoring systems and validated outcome measures (13). For instance, outcome on the Glasgow outcome scale (GOS) was reported for only about a quarter (26.0%) of such group of studies in a recent scoping review (5). This is quite apart from the other fact that many of these publications from the African LMICs touching on cranial neurotrauma are actually on the not-so-well-defined subject of head injury (HI). Thus, with only occasional exceptions, they feature very low brain-CT rates; found to be only 12.0% in the recent review; and the brain imaging findings are also not usually well reported (5, 13).

Drawing on the comparative strength of a prospective registry of HI in our academic neurosurgery practice (14), the aim of this study was to more rigorously characterize the clinical profiles and socio-demographics of TBI in a typical sub-Sahara African LMIC using standardized clinical and radiological diagnostic scoring systems, as well as the in-hospital management-outcome using a validated outcome measure.

## Materials and methods

The data for this study were drawn from a prospectively maintained TBI registry in a sole academic neurosurgery practice in Nigeria. The senior author is one of a faculty of 5 to 6 neurosurgeons in the nation's foremost academic neurosurgery unit. Comprehensive clinical details of all TBI cases managed under his care were consecutively captured in structured clinical summary forms (13), later transferred into electronic spreadsheets forming a prospective registry launched in the year 2009. The senior author is the primary recorder and custodian of the registry, with additional data-acquisition support from rotating neurosurgery resident physicians over the years. The registry is still ongoing. Previous interrogations of this registry have generated earlier publications on related aspects of HI (15–17). Institutional approval (IRB) for the current analysis was obtained from the University of Ibadan/University College Hospital Ethics Committee (UI/EC/25/01211). In this study, all forms of head trauma sparing the brain were excluded; only cases fulfilling the definition of TBI were included, that is, cranial trauma with definite brain pathology, and or perturbation of brain function, qualified and quantified using relevant clinical features [including loss of consciousness (LOC), vomiting, seizures and pupillary anomaly] as well as brain imaging findings.

Specific variables extracted included selected social-clinical demographics (age, gender, TBI etiology), and in-hospital clinical and radiological examination findings; TBI severity was quantified using the Glasgow Coma Scale (GCS) (18, 19) and the Abbreviated Injury Severity-Head (AIS-Head) score (20). Extracranial injuries were graded with the corresponding regional AIS. Overall trauma severity was quantified with the Injury Severity Score (ISS), scaled 1–75, with higher scores denoting greater severity (20). Brain computed tomography (CT) findings were categorized using the Rotterdam CT (CT Rott) scoring system, scaled 1–6, with higher scores indicating more significant TBI (21, 22). Finally, the patients' in-hospital course including the treatment received, surgical or non-surgical, was noted, and the in-hospital outcome was graded using the Glasgow Outcome Scale (GOS), dichotomized as good outcome (good recovery or moderate disability), vs. poor outcome (severe disability, persistent vegetative state, or death) (21, 22).

### Statistical analysis

This arm of this project attempts only descriptive observation please. Hence, only simple descriptive statistical evaluations were carried out; the datawere analyzed using the SPSS version 21 (IBM Corp, Armonk, NY). Findings are presented as frequencies and proportions for categorical variables; mean (Standard deviation, SD) or median (interquartile range, IQR,) for continuous variables depending on the normality or otherwise of the distribution (normality adjudged by the size of the SD). Only few variables showed missing values, and those were also a very negligible proportion in most cases. Nevertheless, analyses of such variables were done using only the available figure as the denominator. The chi-square test was used for associations between categorical variables. A *p*-value < 0.05 was deemed statistically significant.

## Results

Over the 13-year period (2009–2022), 1,440 cases of TBI meeting the study definition were recorded. There were 1,110 males (77.1%) and 330 females (22.9%), a male-to-female ratio of 3.4:1. The cohort was predominantly young and economically active (Figure 1), with a mean age of 32.2 (SD, 19) years old, modal distribution in the 3rd and 4th decades. Only about 10% was beyond the 6th decade.

**Figure 1:**
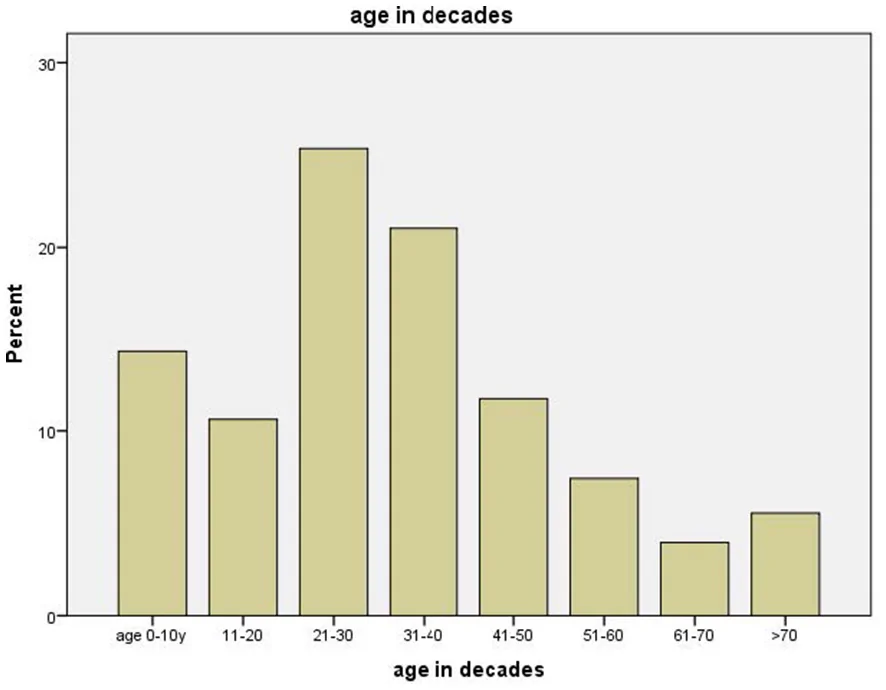
TBI in an LMIC in the CT era: age distribution in decades of the patient population. CT, computed tomography; LMIC, low middle-income country; TBI, traumatic brain injury.

Table 1 outlines other relevant clinical demographics. Road trauma was the TBI etiology in more than four-fifths of cases, and about a third of these were pedestrians. Even in the elderly (>60 years), road trauma accounted for 121/137 (88.3%) cases, while falls accounted for only 12/137 (8.8%). In the pediatric cohort (aged ≤ 14years), falls accounted for 27.4% (73/266) of TBI and road traffic accidents (RTAs) for 65.4% (174/266).

**Table 1:** TBI in an LMIC in the CT era: clinical demographics of the patients.

Variables	Number (%)
Sex (*N* = 1,440)	
Male	1,110 (77.1)
Female	330 (22.9)
Cause of head trauma (*N* = 1,440)	
Road accidents	1,173 (81.5)
Fall	136 (9.4)
Assaults	79 (5.5)
Others	52 (3.6)
Categories of trauma victims (*N* = 1,173)	
Riders/Drivers/Passengers	788 (67.2)
Pedestrians	385 (32.8)
Neurosurgery presentation posttrauma (*N* = 1,439)	
Directly to our unit	345 (24.1)
Private hospitals	703 (48.8)
General/District hospitals	267 (18.5)
Other tertiary hospitals	124 (8.6)
Mode of transfer to Neurosurgery (*N* = 1,440)	
Unknown	278 (19.3)
‘Ambulance'	204 (14.2)
Public/Private vehicles	958 (66.5)

CT, computed tomography; LMIC, low-middle income country; TBI, traumatic brain injury.

Among the 1,173 (81.3%) cases of road trauma, the responsible vehicles were motorcycles (2-wheelers) in 659/1,170 (56.3%), motor vehicles in 42.9%, and tricycles (3-wheelers) in 9/1,170 (1.0%). More than three-quarters had been assessed at outlying health facilities (ranging from primary to tertiary) prior to presentation at our neurosurgery unit, arriving after a median delay of 13 (IQR 4, 48) hours. Although the means of the inter-hospital transport for many of these referrals was unknown, still, as many as two-thirds (66.5%) arrived in private vehicles, sometimes motorcycles.

Clinical evidence of TBI (Table 2) included LOC in 89% of the cases, median duration 11 (IQR 2, 48) hours; along with headache, seizures, otorrhoea/rhinorrhoea and pupillary anomaly.

**Table 2:** TBI in an LMIC in the CT era: clinical presentation.

Symptoms	*N* (%)
Loss of consciousness	1,285 (89.2)
ENT effluxes	526 (36.5)
Vomiting	353 (24.5)
Seizures	228 (15.8)
Headache	222/1,415 (15.7)
Anisocoria	392/1,434 (27.3)

*N* = 1,440 (unless otherwise indicated); Number in parenthesis = %. CT, computed tomography; ENT, ear nose and throat; LMIC, low-middle income country; TBI, traumatic brain injury.

Brain CT scan was obtained in more than a thousand, 1,046/1,440 (72.6%), of cases; and, using the GCS grading, mild TBI (GCS 13–15) occurred in 763/1,440 (53.0%), moderate (GCS 9–12) in 365 (25.3%), and severe (GCS 3–8) in 314 (21.7%). Table 3 summarizes additional severity metrics: AIS-Head > II in 85.7%; and CT-Rotterdam score >2 in 51.5% of those imaged. TBI frequently occurred in the context of other-system injury (involving the maxillofacial regions, pelvis/long bones, thorax and spine), resulting in a mean ISS of 24.5 (SD, 23.2) with 1,019/1,396 (73.0%) having an ISS greater than 16 (22).

**Table 3:** TBI in an LMIC in the CT era: brain injury severity and associated extracranial trauma.

AIS- Head	*N* = 1,396 (%)	CT Rotterdam	*N* = 1,019(%)	Other system trauma	*N* = 1,440 (%)
1	053 (3.8)	1	15 (1.5)	Maxillofacial	460 (31.9)
2	147 (10.5)	2	479 (47.0)	Pelvis/long bones	255 (17.7)
3	248 (17.8)	3	294 (28.9)	Spine	133 (9.2)
4	439 (31.4)	4	105 (10.3)	Chest	102 (7.1)
5	508 (36.4)	5	79 (7.8)	Abdomen	33 (3.0)
6	001 (0.1)	6	47 (4.6)		

AIS, abbreviated injury scale; CT, computed tomography; LMIC, low-middle income country; TBI, traumatic brain injury.

Indications of the treatment given were available for 1430/1440 (99.0%) of patients. Non-operative treatment was employed in 1,149/1,430 (80.3%); 188/1430 (13.1%) underwent definitive operative neurosurgical intervention, the rest either left against medical advice, or had the proposed surgery either declined or canceled for various logistic reasons.

In-hospital outcome data were available for 1,397/1,440 (97.0%). About three-quarter (74.8%, 1,046/1,397) of these made good outcome per dichotomized GOS, Table 4. The 351 (25.1%) with poor outcome included 8.1% (113) patients who left against medical advice at varying stages of TBI recovery. In-hospital deaths totaled 294 (21.1%). Table 5 shows the age distribution of the mortality: 65% occurred in patients aged 20–59 years (3rd through 6th decades), 17% in childhood and adolescence, and 17.7% in patients aged > 60 years. In-hospital outcome was poor in close to 80.0% of patients with severe TBI, majority of whom also incurred significant brain injury on imaging and accounted for nearly two-thirds (64.0%) of the total mortality burden (Table 6).

**Table 4:** TBI in an LMIC in the CT era: in-hospital outcome at discharge or death of the patients.

Glasgow outcome scale *N* = 1,397	Number	(%)
Good recovery	709	50.7
Moderate deficit	337	24.1
Severe deficit	46	3.3
Persistent vegetative state	11	0.8
Death	294	21.1

CT, computed tomography; LMIC, low middle-income country; TBI, traumatic brain injury.

**Table 5:** TBI in an LMIC in the CT era: mortality distribution by age group.

Age in decades	% mortality N-294
0–10y	27(9.2)
11–20	23 (7.8)
21–30	58 (19.7)
31–40	62 (21.1)
41–50	44 (15.0)
51–60	28 (9.5)
61–70	20 (6.8)
>70	32 (10.9)

CT, computed tomography; LMIC, low-middle income country; TBI, traumatic brain injury.

**Table 6:** TBI in an LMIC in the CT era: clinical /radiological severity versus in-hospital outcome.

Severity of TBI using GCS	*N* (%)	(%) CT Rotterdam score > 2	*p*-value	In-hospital outcome good	*p*-value
Mild TBI (GCS 13–15)	763 (53.0)	56.9		644 (84.4)	
Mod TBI (GCS 9–12)	365 (25.3)	69.3		235 (64.4)	
Severe TBI (GCS 3–8)	314 (21.7)	83.0^*^	< 0.01	74 (23.6)^*^	< 0.01

^*^Chi-squared test. CT Rotterdam, computed tomography; GCS, Glasgow coma scale; LMIC, low-middle income country; Mod TBI, moderate TBI; TBI, traumatic brain injury.

## Discussion

In this work, the results of an analysis of a comparatively well-curated database on the subject of TBI from a developing country in the contemporary computerized brain-imaging era are presented. On the one hand, the findings corroborate some of the previously described clinical-epidemiological characteristics of head trauma across many LMICs (8). Traumatic brain injury occurred in all age groups from childhood to the elderly, but most frequently among the young male, mean age 32 years. Road trauma, predominantly by two-wheeler- (motorcycle) crashes, is the culprit in more than 80%, even amongst the elderly patients; a pattern contrasting sharply with Western HICs where falls now predominate among the elderly (3, 4). Thus, efforts to reduce the burden of TBI in developing countries must necessarily continue to be devoted to reducing road trauma. Nonetheless, there is a noteworthy emergence of three-wheeler- (tricycle) crashes as a new cause of neurotrauma; whereas falls had a comparatively much higher proportion in childhood. Nearly a third of the road trauma cases involved pedestrians. Onsite/pre-neurosurgical care was sub-optimal; patients suffered significant associated extracranial trauma; arrived late in private transport; and presented with clinical/radiological evidence of significant brain injury (23). In other words, both the pre-hospital and in-hospital phases of the care of TBI remain at the sub-optimal levels in our system, as may be the case in many other LMICs.

On the other hand, this study presents some unique features distinguishing it from similar ones from comparative geographical regions. Firstly, it more specifically speaks to the subject of TBI by deploying standardized scoring/grading scales, including the GCS, AIS-Head, and even more importantly a high brain CT-acquisition rate (73%) in more than 1,000 patients. For instance, not only was the trauma etiology for the whole cohort outlined, we also outlined the specific etiology in the pediatric, as well as the geriatric age group. Thus we showed the constrating etiology of TBI being road trauma, and not fall, in the aged patients, in contrast to what obtains in most Western countries in the current era.

The brain CT scan findings were also characterized with a standardized scoring system, the CT – Rotterdam grading. This is unlike most contemporaneous works from global regions with similar low human development index (HDI) which only address the less well-defined subject of HI, many times by non-neurosurgeons (24, 25), and usually with only very low rate, if at all, of computerized brain imaging (8). One earlier report on HI from our stable featured brain CT rate of a mere 28% (26). A more recent work from a rural practice 29% (27); and a pediatric TBI paper from Uganda featured only 40% cranial imaging, which actually included skull radiography (X-ray) and CT scanning (28). In the same token, two recent, comparable registry-based studies from two other African LMICs, Uganda and Tanzania, similarly grappled with resource constraints limiting access to diagnostic imaging (29–31).

The high CT-acquisition rate in this study reflects some institutional advantages in our practice (32). Firstly, ours is the flagship neurosurgery unit in all of the sub-Saharan Africa, and sits at the apex of medical care in our nation's foremost university teaching hospital. Thus, many patients referred to us from outlying health facilities arrive accompanied by their brain CT imaging. In addition, we periodically benefit from highly subsidized trauma CT-scanning in-house, facilitating timely neuroimaging for patients who would otherwise be unable to afford it (33).

On another note, this study showed that even in a tertiary center receiving predominantly referred and more severe cases, majority of TBI (close to 80%) were deemed non-surgical (34). Hence, only 13% of the patient-cohort received operative intervention. The details of these surgical procedures shall be presented in a later report. It has actually been once suggested that patients with mild/moderate TBI appear to make better in-hospital outcome in regions of the world with lower healthcare resources than comparable cohorts in HICs (24, 34, 35). The reasons for this intriguing observation remain speculative but differential case-mix, referral patterns and reporting definitions may all contribute.

Thus, among the 1,397/1,440 (97%) of this patient cohort in which in-hospital outcome data were available, up to 74.8% made good outcome. This outcome was associated with the severity of the TBI clinically and radiologically. Close to 300 patients (21%) died, 65% of them in their 3rd to 6th decades of life, another 17% in childhood/adolescence. This is such an unfortunate wastage of the very active and productive members of the LMICs population, the breadwinners of their respective families in regions already bogged down by low metrics on the HDI. This heavy toll on precious human life surely underscores the urgent imperative to curtail the perennially untrammeled road carnage across Africa and all of the LMICs (9).

This study has some notable limitations. As a single-surgeon series from a single institution at the apex of a national referral chain, the patient cohort is necessarily highly selected and cannot be considered representative of the typical spectrum of TBI in the broader Nigerian population. There is also the exclusive use of in-hospital outcomes without long-term follow-up, as well as the absence of detailed analyses of surgically treated patients. This calls for caution in viewing the findings regarding the outome of TBI in this part of the world. Also, a prolonged data acquisition, spanning more than a decade, may introduce temporal heterogeneity in database quality, a concern particularly relevant during the COVID-19 pandemic when registry entries declined substantially. In the same token, changes in referral patterns, imaging availability, treatment strategies, and overall trauma care over more than a decade might have influenced the results.

Conversely though, the extended study duration might as a matter of fact be a methodological strength: it ensures capture of epidemiological trends that would be invisible in shorter, cross-sectional efforts. Also, the prospective design and data acquisition by an experienced neurosurgical team permitted more rigorous characterization of both the clinical and radiological dimensions of TBI that is not typical of reports from this and similar regions of the world. There is a notable comparative work from the neighboring country of Cameroon; a well-funded, multi-center, 5-year retrospective study. The study reported a brain CT-acquisition rate of 67%, somewhat below ours. It characterized brain injuries in reasonable detail, but without the application of any CT-based grading scale; and, it included cases of chronic subdural haematoma (CSDH) within its cohort (36). We contend that CSDH represents a distinct entity from acute TBI and should be considered separately.

The manageably robust nature of the database in our registry also affords opportunities to interrogate for publications in the future many other aspects of the subject of TBI in an LMIC, including a more nuanced analytical study of the determinants of outcomes; sub-group analysis of pediatric and geriatric TBI; brain imaging of TBI in a resource-constrained setting; as well as, among others, detailed characterization of the operative surgery of TBI in an LMIC in the CT era.

## Conclusion

Traumatic brain injury continues to exact a heavy and disproportionate burden in the developing countries claiming precious lives predominantly among the societies' productive segments. As suggested by this single-institution series, its clinical and epidemiological profile differs substantially from that of the high-income countries, with road traffic trauma, particularly involving motorcycles as the dominant mechanism. Significant gaps and inadequacies persist across many aspects of the TBI management continuum particularly from the scene of injury and prehospital phase through the in-hospital acute care (37). These deficiencies represent clearly identifiable opportunities for targeted, evidence-based interventions to improve the current sub-optimal outcome-metrics of TBI in sub-Sahara African LMICs and to fulfill the global commitment to halving road traffic deaths by the year 2030.

## Data Availability

The raw data supporting the conclusions of this article will be made available by the authors, without undue reservation.
